# Beneficial Effects of Galectin-3 Blockade in Vascular and Aortic Valve Alterations in an Experimental Pressure Overload Model

**DOI:** 10.3390/ijms18081664

**Published:** 2017-07-31

**Authors:** Jaime Ibarrola, Ernesto Martínez-Martínez, J. Rafael Sádaba, Vanessa Arrieta, Amaia García-Peña, Virginia Álvarez, Amaya Fernández-Celis, Alicia Gainza, Patrick Rossignol, Victoria Cachofeiro Ramos, Natalia López-Andrés

**Affiliations:** 1 Cardiovascular Translational Research, Navarrabiomed (Miguel Servet Foundation), Instituto de Investigación Sanitaria de Navarra (IdiSNA), Pamplona 31008, Spain; jaime.ibarrola.ulzurrun@navarra.es (J.I.); ernesto_mart@hotmail.com (E.M.-M.); jr.sadaba.sagredo@navarra.es (J.R.S.); varriet8@hotmail.com (V.Á.); amaiagpu@hotmail.com (A.G.-P.); virginia.alvarez.asiain@navarra.es (V.A.); amaya.fernandez.decelis@navarra.es (A.F.-C.); alicia.gainza.calleja@navarra.es (A.G.); 2 Institut National de la Santé et de la Recherche Médicale (INSERM), Centre d’Investigations Cliniques-Plurithématique 1433, UMR 1116 Université de Lorraine, CHRU de Nancy, French-Clinical Research Infrastructure Network (F-CRIN) INI-CRCT, Nancy 54500, France; p.rossignol@chu-nancy.fr; 3 Department of Physiology, School of Medicine, Instituto de Investigación Sanitaria Gregorio Marañón (IiSGM), Universidad Complutense, Madrid 28040, Spain; vcara@ucm.es

**Keywords:** Galectin-3, pressure overload, aorta, valve

## Abstract

Galectin-3 (Gal-3) is involved in cardiovascular fibrosis and aortic valve (AV) calcification. We hypothesized that Gal-3 pharmacological inhibition with modified citrus pectin (MCP) could reduce aortic and AV remodeling in normotensive rats with pressure overload (PO). Six weeks after aortic constriction, vascular Gal-3 expression was up-regulated in male Wistar rats. Gal-3 overexpression was accompanied by an increase in the aortic media layer thickness, enhanced total collagen, and augmented expression of fibrotic mediators. Further, vascular inflammatory markers as well as inflammatory cells content were greater in aorta from PO rats. MCP treatment (100 mg/kg/day) prevented the increase in Gal-3, media thickness, fibrosis, and inflammation in the aorta of PO rats. Gal-3 levels were higher in AVs from PO rats. This paralleled enhanced AV fibrosis, inflammation, as well as greater expression of calcification markers. MCP treatment prevented the increase in Gal-3 as well as fibrosis, inflammation, and calcification in AVs. Overall, Gal-3 is overexpressed in aorta and AVs from PO rats. Gal-3 pharmacological inhibition blocks aortic and AV remodeling in experimental PO. Gal-3 could be a new therapeutic approach to delay the progression and the development of aortic remodeling and AV calcification in PO.

## 1. Introduction

Chronic pressure overload (PO) results in pathological morphological changes in the cardiovascular system. These changes result in an initially compensatory phase, but if they persist, could produce an important impact on cardiovascular function. PO could be due to aortic stenosis (AS) and may promote heart failure (HF) [1]. Of note, AS is the most common heart valve disease (43.1%) and represents a major healthcare burden [2]. At the cardiac level, PO induces cardiac hypertrophy [3]. At the vascular level, PO initially leads to an increase in aortic diameter and thickening of the aortic wall. Remodeling is characterized by an inflammatory phase accompanied by matrix modifications resulting in fibrosis. At the vascular level, this fibrosis could modify the diameter of the vessels and promote arterial stiffness, a change known to be predictive of increased cardiovascular mortality [4,5]. However, the molecular signaling pathways responsible for vascular remodeling in PO have not been identified.

PO also induces a modification in the aortic valves (AVs). The valve cusps become progressively thickened, fibrosed, and calcified [6]. A combination of endothelial damage and lipid deposition triggers inflammation within the AV that allows infiltration of inflammatory cells which release proinflammatory cytokines [7,8,9]. In addition, matrix metalloproteinases (MMPs) secreted by valvular interstitial cells (VICs) and inflammatory cells have an important and complex role in the restructuring of the AV matrix [8]. As the stenosis-induced PO progresses, wall shear stress across the aortic valve dramatically increases [4], activating transforming growth factor (TGF)-β1 [5], which can also induce fibrosis and calcification [10]. It is also known that shear stress is able to modulate factors involved in vascular remodeling [9]. Elucidation of the potential mechanisms underlying vascular remodeling and AV calcification could provide new biotargets and new pharmacological approaches for the treatment of vascular and valvular diseases.

Galectin-3 (Gal-3) is a 29 to 35 kDa protein and member of a β-galactoside binding lectin family, which interacts with cell surface receptors and extracellular matrix (ECM) proteins [10]. Gal-3 levels are increased in myocardial biopsies and AVs from AS patients [11,12,13]. In addition, patients with high Gal-3 plasma levels are associated with adverse outcome after trans-catheter AV implantation, suggesting that Gal-3 could play an important role in patients with AS [14]. In vitro, Gal-3 induces inflammation, fibrosis, and calcification in VICs, and at the vascular level, Gal-3 plays an important role in increasing ECM components in vascular smooth muscle cells (VSMCs) [12,15]. These results suggest a key role for Gal-3 in cardiac alterations in PO conditions. Ascending aortic constriction is the most common surgical model for creating PO induced cardiac alterations. A major advantage of the model is the gradual onset of PO on the heart and the associated structural changes such as cardiac hypertrophy and remodeling [3]. In a recent study, we confirmed the role of Gal-3 in early cardiac molecular alterations associated with PO in normotensive rats subjected to aortic banding. In PO conditions, cardiac Gal-3 expression is augmented, and its pharmacological inhibition prevented cardiac fibrosis and inflammation [13]. We have demonstrated that Gal-3 inhibition prevented vascular remodeling in different pathologies such as aldosterone excess [16] and obesity [13,17]. In the present study, we would like to extend the knowledge of the role of Gal-3 at both the vascular and valvular levels in PO conditions. Therefore, our hypothesis is that besides its protective cardiac effects, Gal-3 inhibition could delay vascular remodeling and AV inflammation, fibrosis, and calcification.

## 2. Results

### 2.1. Aortic Structural Changes Are Improved by Galectin-3 (Gal-3) Blockade in Pressure Overload Rats

Animals subjected to partial occlusion of ascending aorta presented similar body weight (BW) and blood pressure as compared to control rats [12] after six weeks.

**Figure 1:**
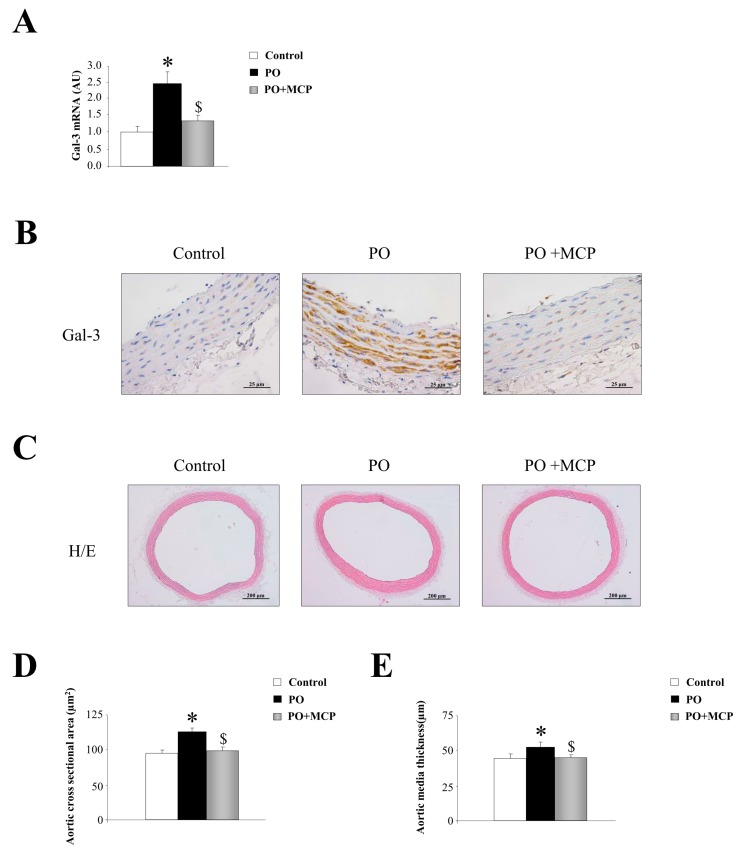
Pharmacological inhibition of Galectin-3 (Gal-3) decreases aortic wall thickness. Quantification of aortic Gal-3 mRNA levels in controls, pressure overload (PO) and PO + modified citrus pectin (MCP) rats (**A**); representative pictures of aortic sections immunostained for Gal-3 (**B**); haematoxylin/eosin staining of aortas (**C**); and quantification of aortic morphometry (**D**,**E**). Histogram bars represent the mean ± standard error of mean (SEM) of each group of rats in arbitrary units normalized to hypoxanthine phosphoribosyltransferase (HPRT) and β-actin for cDNA. Magnification 40×. (Control rats, *n* = 7; Pressure Overload rats, PO, *n* = 7; and Pressure Overload rats treated with MCP, PO + MCP, *n* = 7). * *p* < 0.05 vs. control group; ^$^ *p* < 0.05 vs. PO group.

Gal-3 mRNA expression was increased in aorta from PO rats as compared to that of controls, and was normalized by the treatment with the Gal-3 inhibitor modified citrus pectin (MCP) (Figure 1A). This result was confirmed by immunohistochemistry, showing that the increase in Gal-3 immunostaining presented in PO aortas was reduced by MCP treatment (Figure 1B). Haematoxylin/eosin staining of the aortas is shown in Figure 1C. At the histological level, aortas from PO rats exhibited increased media cross sectional area (Figure 1D) as well as increased media thickness (Figure 1E) as compared to that of controls. All aortic morphological changes were normalized by MCP treatment (Figure 1D,E).

### 2.2. Gal-3 Pharmacological Blockade Diminishes Aortic Remodeling in Pressure Overload (PO) Rats

**Figure 2:**
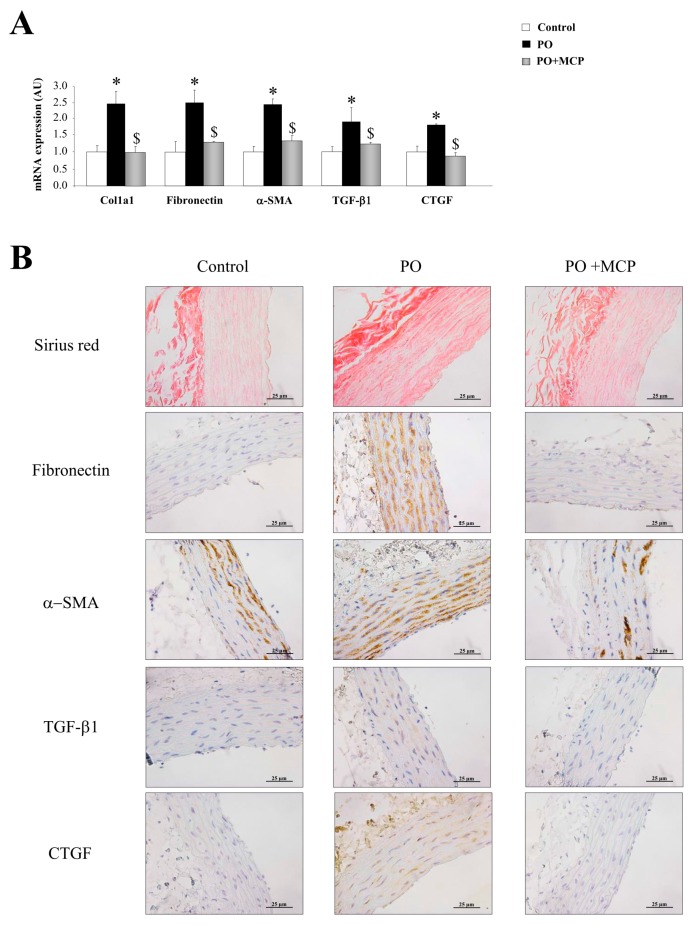
Pharmacological inhibition of Gal-3 prevents vascular fibrosis. Quantification of mRNA levels of Col1a1, fibronectin, α-smooth muscle actin (α-SMA), transforming growth factor (TGF)-β1, and connective tissue growth factor (CTGF) in aorta from controls, PO and PO + MCP rats (**A**); representative pictures of aortic sections immunostained for Sirius red, fibronectin, α-SMA, TGF-β1, and CTGF (**B**). Histogram bars represent the mean ± SEM of each group of rats in arbitrary units normalized to HPRT and β-actin for cDNA. Magnification 40×. (Control rats, *n* = 7; Pressure Overload rats, PO, *n* = 7; and Pressure Overload rats treated with MCP, PO + MCP, *n* = 7). * *p* < 0.05 vs. control group; ^$^ *p* < 0.05 vs. PO group.

The PO group presented an increase in aortic collagen type I (Col1a1) (*p* < 0.05), fibronectin (*p* < 0.05), α-smooth muscle actin (α-SMA) (*p* < 0.05), TGF-β1 (*p* < 0.05), and connective tissue growth factor (CTGF) (*p* < 0.05) mRNA levels as compared to that of controls (Figure 2A). MCP treatment was able to reduce the increase in profibrotic molecules analyzed in PO animals (Figure 2A). In accordance with these observations, PO animals presented higher total collagen as well as increased fibronectin, α-SMA, TGF-β1, and CTGF immunostainings (Figure 2B). Gal-3 inhibition completely reversed this (Figure 2B).

**Figure 3:**
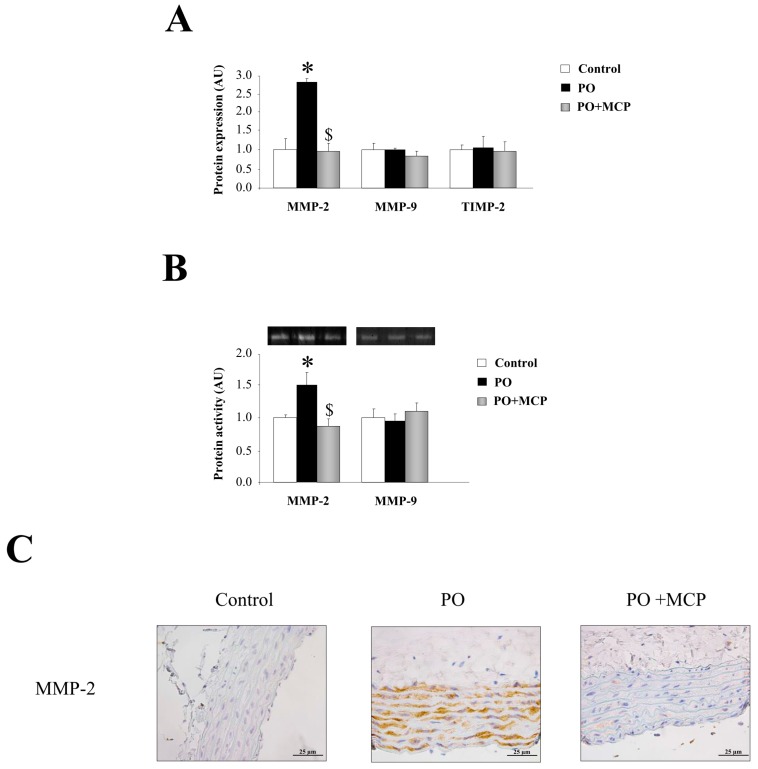
Pharmacological inhibition of Gal-3 blocks matrix metalloproteinase (MMP)-2 activity. Normalized values of the protein levels of MMP-2 (ng/mL), MMP-9 (pg/mL), and tissue inhibitor of metalloproteinase (TIMP)-2 (pg/mL) in aorta from controls, PO, and PO + MCP rats measured by ELISA (**A**); MMPs activities assessed by zymography (**B**); Representative pictures of aortic sections immunostained for MMP-2 (**C**). Histogram bars represent the mean ± SEM of each group of rats in arbitrary units. Magnification 40×. (Control rats, *n* = 7; Pressure Overload rats, PO, *n* = 7; and Pressure Overload rats treated with MCP, PO + MCP, *n* = 7). * *p* < 0.05 vs. control group; ^$^ *p* < 0.05 vs. PO group.

Complementary, aortas from PO rats presented higher MMP-2 expression (*p* < 0.05) as compared to that of controls (Figure 3A). MCP treatment blocked aortic MMP-2 increase. MMP-9 and tissue inhibitor of metalloproteinase (TIMP)-2 levels were similar in aortas from the three groups of animals (Figure 3A). This result was confirmed by zymography, showing that MMP-2-increased activity in aortic wall from PO animals (*p* < 0.05) was normalized by MCP treatment (Figure 3B). Finally, MMP-2 immunostaining was higher in aorta from PO rats as compared to controls and PO + MCP animals (Figure 3C).

### 2.3. Inhibition of Gal-3 Attenuates Vascular Inflammation in PO Rats

**Figure 4:**
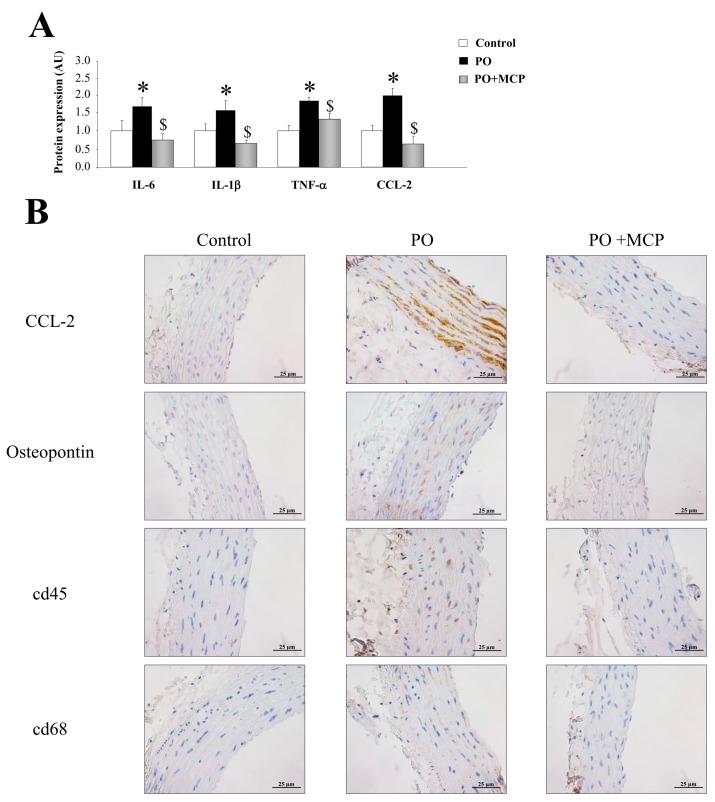
Pharmacological inhibition of Gal-3 reduces vascular inflammation. Normalized values of the protein levels of interleukin (IL)-6 (pg/mL), IL-1β (pg/mL), tumor necrosis factor (TNF)-α (pg/mL), and monocyte chemoattractant protein (CCL)-2 (pg/mL) in aorta from controls, PO, and PO + MCP rats using ELISA (**A**); representative pictures of aortic sections immunostained for CCL-2, osteopontin, cd45, and cd68 (**B**). Histogram bars represent the mean ± SEM of each group of rats in arbitrary units. Magnification 40×. (Control rats, *n* = 7; Pressure Overload rats, PO, *n* = 7; and Pressure Overload rats treated with MCP, PO + MCP, *n* = 7). * *p* <0.05 vs. control group; ^$^ *p* < 0.05 vs. PO group.

Vascular expression of interleukin (IL)-6, IL-1β, tumor necrosis factor (TNF)-α, and monocyte chemoattractant protein-1 (CCL-2) was higher (*p* < 0.05) in PO group (Figure 4A). Moreover, aortas from PO rats presented higher staining for CCL-2 and osteopontin, as well as for cd45 and cd68 (Figure 4B). MCP treatment reduced the increase in inflammatory markers observed in PO aortas (Figure 4A,B).

### 2.4. Gal-3 Blockade Reduces Aortic Valve (AV) Thickening and Fibrosis in PO Rats

**Figure 5:**
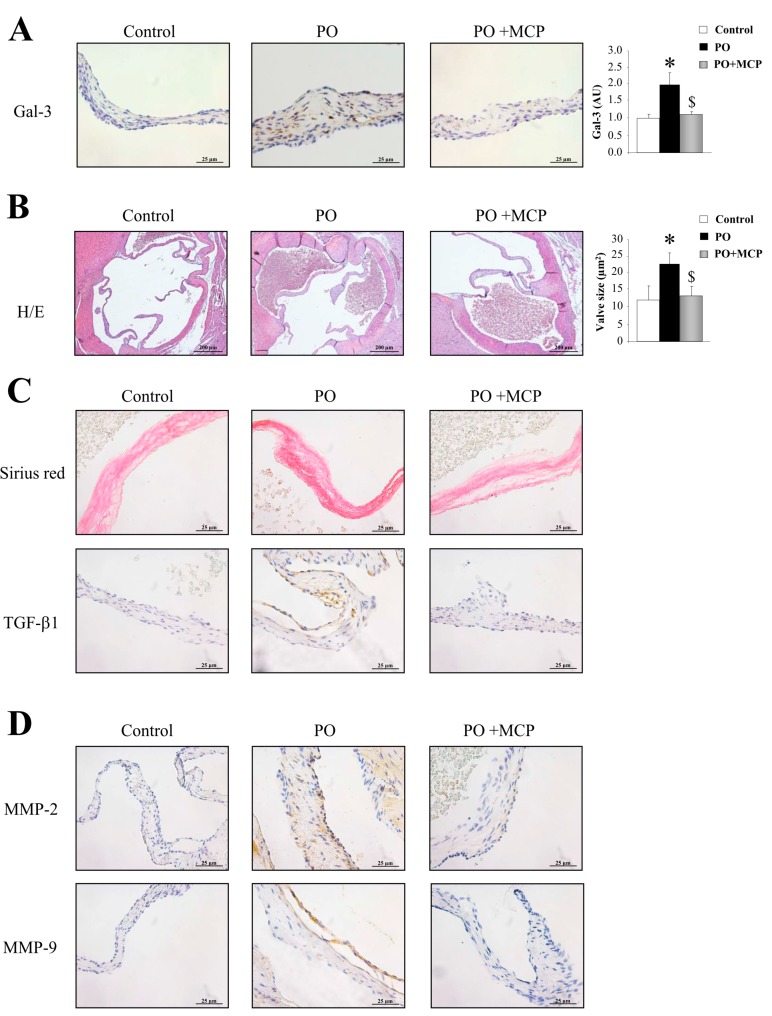
Pharmacological inhibition of Gal-3 diminishes aortic valve (AV) fibrosis. Immunostaining and quantification for Gal-3 in AVs from controls, PO, and PO + MCP rats (**A**). Haematoxylin/eosin staining of AVs and quantification of AV area (**B**); Sirius red staining and TGF-β1 immunostaining in AVs (**C**); MMP-2 and MMP-9 immunostaining in AVs (**D**). Histogram bars represent the mean ± SEM of each group of rats. Magnification 40×. (Control rats, *n* = 7; Pressure Overload rats, PO, *n* = 7; and Pressure Overload rats treated with MCP, PO + MCP, *n* = 7). * *p* < 0.05 vs. control group; ^$^ *p* < 0.05 vs. PO group.

Gal-3 expression was increased in AVs from PO rats as compared to that of controls (*p* < 0.05), and was normalized by MCP treatment (Figure 5A). Haematoxylin/eosin staining was used to analyze the microstructure of the AVs obtained from control, PO, and PO + MCP rats (Figure 5B). As shown in the microphotographs, AV from PO rats exhibited increased AV areas as compared to that of controls and PO + MCP rats (*p* < 0.05). Moreover, PO animals presented higher staining for total collagen as well as for TGF-β as compared to that of controls and PO rats receiving MCP (Figure 5C). MMP-2 and MMP-9 immunostaining was greater in AVs from PO rats as compared to that of controls and PO rats receiving MCP (Figure 5D).

### 2.5. Gal-3 Inhibition Decreases AV Inflammation and Calcification in PO Rats

**Figure 6:**
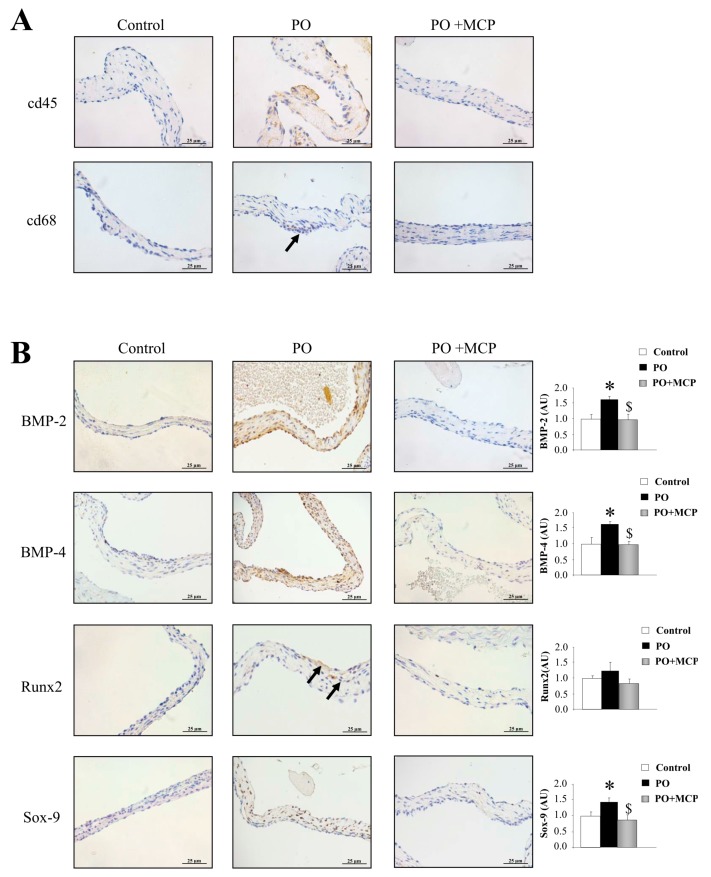
Pharmacological inhibition of Gal-3 reduces AV inflammation and calcification. Representative pictures of AV sections immunostained for cd45 and cd68 (**A**); Immunostaining (left panel) and quantification (right panel) for bone morphogenetic protein (BMP)-2, BMP-4, Runx2, and SRY-homeobOX-like (Sox-9, SRY: *Sex-region-determining Y* gene) in AVs from controls, PO, and PO + MCP rats (**B**). Arrows show the staining. Histogram bars represent the mean ± SEM of each group of rats. Magnification 40×. (Control rats, *n* = 7; Pressure Overload rats, PO, *n* = 7; and Pressure Overload rats treated with MCP, PO + MCP, *n* = 7). * *p* < 0.05 vs. control group; ^$^ *p* < 0.05 vs. PO group.

The immunostaining for leucocytes (cd45) and macrophages (cd68) was higher in AVs from PO rats as compared to that of controls and PO + MCP rats (Figure 6A). The quantification of the expression of the calcification markers revealed that AVs from PO rats presented higher bone morphogenetic protein (BMP)-2 (*p* < 0.05), BMP-4 (*p* < 0.05), and SRY-homeobOX-like (Sox)-9 (*p* < 0.05, *SRY*: *Sex-region-determining Y* gene) (Figure 6B). Runx2 levels were slightly (but not significantly) increased in PO rats as compared to that of controls. MCP treatment diminished the expression of all the calcification markers analyzed.

## 3. Discussion

The aim of this study was to evaluate the effects of Gal-3 inhibition on vascular and AV alterations in an experimental normotensive model PO. In this model, Gal-3 inhibition was shown to exhibit anti-fibrotic and anti-inflammatory properties in the myocardial tissue [13]. Our results demonstrate that Gal-3 inhibition exerts beneficial effects, decreasing aortic tunica media hypertrophy, fibrosis, and inflammation as well as diminishing AV fibrosis, inflammation, and calcification. Taken together, these findings reveal a role for Gal-3 in vascular and valvular alterations associated with PO.

Partial occlusion of the ascending aorta is a common experimental method to induce PO, cardiac hypertrophy, and HF. Although previous studies have shown thickening of the aortic wall, adventitial hyperplasia, collagen deposition, and inflammatory changes, the molecular signaling pathways responsible for this remodeling have not been identified [18,19,20,21,22,23]. Importantly, Gal-3 overexpression increased the collagen type I protein levels in VSMCs, and Gal-3 blockade reduces aortic fibrosis and inflammation in other pathological conditions showing its key role in vascular damage [16]. Moreover, Gal-3 promotes the vascular calcification associated with atherosclerosis [15]. Interestingly, Gal-3 positively associated with pulse wave velocity, an index of arterial stiffness in a community sample [24]. The authors suggest that Gal-3 may contribute toward adverse cardiovascular effects in-part through an effect on aortic stiffness, effects which cannot be attributed to generalized inflammation. Moreover, Gal-3 contributes to ventricular-vascular uncoupling in HF patients [25]. In line with these findings, Gal-3 blockade improved vascular remodeling in PO animals, diminishing vascular hypertrophy, fibrosis, and inflammation. Moreover, Gal-3 blockade does not affect vascular apoptosis (in preparation). In previous studies from our group, we have demonstrated the beneficial effects of Gal-3 inhibition in vascular fibrosis and inflammation in different models associated with hypertension [13,16,17,26,27]. In this study, we used an animal model without modifications in blood pressure [12] in order to avoid any potential confounding factors on fibrosis and inflammation. The fact that Gal-3 inhibition decreased vascular infiltration of inflammatory cells and the fibrotic process in different pathological conditions [13,16,17,26,27] suggests a common mechanism even in the absence of blood pressure modifications.

Gal-3 expression has been recently reported in VICs from AVs in patients undergoing AV replacement [12]. Moreover, Gal-3 enhanced inflammatory, fibrotic, and osteogenic markers in VICs and co-localized with the expression of osteogenic and inflammatory markers in human AVs [12]. Furthermore, we have shown that in vitro, in human VICs, Gal-3 pharmacological inhibition with MCP as well as Gal-3 silencing attenuated the pro-inflammatory, pro-fibrotic, and pro-osteogenic response [12]. A recent study described an association of Gal-3 with mortality after balloon aortic valvuloplasty, which is indicative of a contribution of local valvular Gal-3 expression to post-valvuloplasty restenosis [28]. In PO, there is evidence of aberrant matrix deposition and valve fibrosis, which contributes to the calcification [29]. Abnormal remodeling in the AVs is also accompanied by the deregulated expression of MMPs and inflammation [30]. In agreement with these data, PO animals presented an increase in AV inflammation, fibrosis, MMP activities, and calcification markers. The pharmacological inhibition of Gal-3 was able to decrease the AV inflammation, fibrosis, MMP activities, and calcification in absence of increased blood pressure levels in the PO group, showing the potential therapeutic benefit of Gal-3 inhibition both in the primary (i.e., in early stages of PO) and secondary prevention settings (i.e., when PO is installed).

In conclusion, our findings demonstrate that Gal-3 pharmacological blockade delays alterations in aorta and AVs in PO rats. These results indicate that Gal-3 may be a new potential biotarget for delaying aortic remodeling and AV inflammation and calcification in PO conditions.

## 4. Materials and Methods

### 4.1. Animals

Adult male Wistar rats were obtained from Harlan Ibérica (Barcelona, Spain). Rats were subjected to PO by placing a clip in the ascending aorta as previously described [19,20,21,22,23,31]. Rats were distributed in three different groups: Control rats (sham operated) (Control; *n* = 7), PO rats (PO; *n* = 7), and PO rats receiving the Gal-3 inhibitor modified citrus pectin (PO + MCP; 100 mg/kg/day; *n* = 7) in the drinking water. Treatment was initiated one day prior to the occlusion of the aorta and continued for another six weeks afterwards until the animals were finally sacrificed. The Animal Care and Use Committee of Universidad Complutense de Madrid and Dirección General de Medio Ambiente, Comunidad de Madrid (PROEX 242/15) approved all experimental procedures (12 April 2012) according to the Spanish Policy for Animal Protection RD53/2013, which meets the European Union Directive 2010/63/UE (approval ID: CEA-UCM 77/2012).

### 4.2. Histological Analysis

The ascending aortic section containing the AV and a section of thoracic aorta were dissected. Tissue staining was performed on transversal sections of AVs and aorta. Samples were dehydrated, embedded in paraffin and cut in 5 μm-thick sections.

Slides were treated with H_2_O_2_ for 10 min to block peroxidase activity. All sections were blocked with 5% normal goat serum in phosphate buffered saline (PBS) for 1 h and incubated overnight with Gal-3 (1:1200; Santa Cruz Biotechnology, Dallas, TX, USA; sc-374253), fibronectin (1:50; Santa Cruz Biotechnology; sc-8422), TGF-β (1:200; Santa Cruz Biotechnology; sc-146), CCL-2 (1:100; Santa Cruz Biotechnology; sc-377082), osteopontin (1:600; Santa Cruz Biotechnology; sc-21742), MMP-2 (1:100; Santa Cruz Biotechnology; sc-8835), MMP-9 (1:10; Santa Cruz Biotechnology; sc-6841), cd68 (1:50; Abcam, Hong Kong, China; Ab201340), cd45 (1:100; Santa Cruz Biotechnology; sc-25590), BMP-2 (1:50; Abcam; Ab14933), BMP-4 (1:100; Abcam; Ab39973), Runx2 (1:50; Sigma, St. Louis, MO, USA; AMAb90591), Sox-9 (1:50; Sigma; AMAb90765), α-SMA (1:8000; Sigma; A7607), and CTGF (1:100; Abcam; Ab6992), washed three times, and then incubated for 30 min with the horseradish peroxidase-labeled polymer conjugated to secondary antibodies (Dako Cytomation, Carpentaria, CA, USA). The signal was revealed by using a 3,3′-Diaminobenzidine (DAB) Substrate Kit (BD Pharmingen, San Jose, CA, USA).

For the staining, the slides were hydrated and incubated for 2 h with haematoxylin and eosin staining and 1 h with picrosirius red. After incubations, samples were washed three times and dehydrated.

All measurements and quantifications were performed blind in an automated image analysis system (Nikon, Melville, NY, USA).

### 4.3. Real-Time Reverse Transcription Polymerase Chain Reaction (PCR)

Total RNA from a section of thoracic aorta was extracted with Trizol Reagent (Euromedex) and purified using the RNeasy kit, according to the manufacturer’s instructions (Qiagen, Hilden, Germany). First strand cDNA was synthesized according to the manufacturer’s instructions (Roche, Basel, Switzerland). Quantitative polymerase chain reaction analysis was then performed with *N*′,*N*′-dimethyl-*N*-[4-[(E)-(3-methyl-1,3-benzothiazol-2-ylidene)methyl]-1-phenylquinolin-1-ium-2-yl]-*N*-propylpropane-1,3-diamine (SYBR) green PCR technology (ABGene, Portsmouth, NH, USA) (Table S1). Relative quantification was achieved with MyiQ (Bio-Rad, Hercules, CA, USA) software according to the manufacturer’s instructions. Data were normalized by hypoxanthine phosphoribosyltransferase (HPRT) and β-actin levels and expressed as percentage relative to controls. All PCRs were performed at least in triplicate for each experimental condition.

### 4.4. ELISA

IL-6, IL-1β, TNF-α, CCL-2, MMP-2, MMP-9, and tissue inhibitor of metalloproteinase (TIMP)-2 concentrations were measured in aortic extracts by ELISA according to the manufacturer’s instructions (R&D Systems, Minneapolis, MN, USA).

### 4.5. Gelatin Zymography

Aliquots of aortic tissue containing 15 μg of proteins were resolved on a 10% SDS polyacrylamide gel containing 0.3% gelatin. The gel was rinsed three times for 15 min with a solution of 2.5% Triton × 100 to remove SDS and renature the proteins, followed by incubation for 48 h at 37 °C in 1000 mmol/L Tris-HCl, pH 7.5 with 1000 mmol/L CaCl_2_ and 5000 mmol/L NaCl to promote degradation of gelatin. Gels were fixed in 40% methanol and 10% acetic acid and then stained for 30 min in 0.25% Coomassie blue R-250 to identify proteolytic activity of MMPs.

### 4.6. Statistical Analysis

Data are expressed as mean ± SEM. Normality of distributions was verified by means of the Kolmogorov–Smirnov test. Data were analyzed using a one-way analysis of variance, followed by a Newman-Keuls to assess specific differences among groups or conditions. All analyses was done with SPSS version 20.0 (IBM SPSS Statistics) and amtwo-tailed *p*-value of <0.05 was considered statistically significant.

## 5. Conclusions

Gal-3 pharmacological inhibition blocks aortic and AV remodeling in experimental PO. Gal-3 emerges a new therapeutic approach to delay the progression and the development of aortic remodeling and AV calcification in PO.

## Supplementary Materials

Supplementary materials can be found at www.mdpi.com/1422-0067/18/8/1664/s1.
