# Supplementary material for: Beneficial Effects of Galectin-3 Blockade in Vascular and Aortic Valve Alterations in an Experimental Pressure Overload Model
Source: Int J Mol Sci. 2017 Jul 31;18(8):1664. doi: 10.3390/ijms18081664 (PMC5578054; doi:10.3390/ijms18081664)
Supplement: Supplementary file 1 [file ijms-18-01664-s001.pdf]

Beneficial Effects of Galectin-3 Blockade in Vascular and Aortic Valve Alterations in an Experimental Pressure Overload Model

Table S1: Primers used in rats in real time PCR analysis.

Gene	Primer	Sequence (5´to 3´)
Gal-3 (NC_000014)	Forward	AGC CCA ACG CAA ACA GTA TC
	Reverse	GGC TTC AAC CAG GAC CTG TA
Fibronectin (NC_030685)	Forward	GGG GTC ACG TAC CTC TTC AA
	Reverse	TGG AGG TTA GTG GGA GCA TC
α-SMA (NC_000010)	Forward	GAA GGA ATA GCC ACG CTC AG
	Reverse	TGT GCT GGA CTC TGG AGA TG
Col 1a1 (NC_000017)	Forward	GCC TCC CAG AAC ATC ACC TA
	Reverse	ATG TCT GTC TTG CCC CAA GT
TFG-β (NC_000019)	Forward	CAG AAG TTG GCA TGG TAG CC
	Reverse	TGC TTC AGC TCC ACA GAG AA
CTGF (NC_000006)	Forward	GAG TCG TCT CTG CAT GGT CA
	Reverse	CCA CAG AAC TTA GCC CGG TA
HPRT (NC_000023)	Forward	AGG ACC TCT CGA AGT GT
	Reverse	ATT CAA ATC CCT GAA GTA CTC AT
β-actin (NC_000007)	Forward	CCT CTA TGC CAA CAC AGT GCT GTC T
	Reverse	GCT CAG GAG GAG CAA TGA TCT TGA
